# Genomic signatures of drift and selection driven by predation and human pressure in an insular lizard

**DOI:** 10.1038/s41598-021-85591-x

**Published:** 2021-03-17

**Authors:** Marta Bassitta, Richard P. Brown, Ana Pérez-Cembranos, Valentín Pérez-Mellado, José A. Castro, Antònia Picornell, Cori Ramon

**Affiliations:** 1 Laboratori de Genètica, Departament de Biologia, Universitat de les Illes Balears, Crta. de Valldemossa, km 7.5, 07122 Palma de Mallorca, Spain; 2 School of Biological and Environmental Sciences, Liverpool John Moores University, Liverpool, UK; 3 Departamento de Biología Animal, Edificio de Farmacia, Universidad de Salamanca, Campus Miguel de Unamuno, Salamanca, Spain

**Keywords:** Ecology, Evolution, Genetics

## Abstract

Genomic divergence was studied in 10 small insular populations of the endangered Balearic Islands lizard (*Podarcis lilfordi*) using double digest restriction-site associated DNA sequencing. The objectives were to establish levels of divergence among populations, investigate the impact of population size on genetic variability and to evaluate the role of different environmental factors on local adaptation. Analyses of 72,846 SNPs supported a highly differentiated genetic structure, being the populations with the lowest population size (Porros, Foradada and Esclatasang islets) the most divergent, indicative of greater genetic drift. Outlier tests identified ~ 2% of loci as candidates for selection. Genomic divergence-Enviroment Association analyses were performed using redundancy analyses based on SNPs putatively under selection, detecting predation and human pressure as the environmental variables with the greatest explanatory power. Geographical distributions of populations and environmental factors appear to be fundamental drivers of divergence. These results support the combined role of genetic drift and divergent selection in shaping the genetic structure of these endemic island lizard populations.

## Introduction

Insular populations are naturally isolated systems that harbour high levels of biodiversity and endemism^1^. Their characteristic isolation leads to a reduction in gene flow and generates population divergence and speciation^2^. High levels of genetic structuring also result from frequent physical events combined with the impact of rapid fixation rates in often small populations subject to genetic drift and selection^3,4^.

Understanding the relative roles of selection and drift are key to understanding the divergence of insular populations. Drift is expected to be considerable due to low migration rates and small population sizes^5–7^. Nonetheless, morphological divergence and environmental heterogeneity between islands suggests that divergent selection may also play a key role^8–10^. The interplay between local adaptation and genetic drift in moulding variation in these environments is often not clear and requires more research^11–13^. Genetic and genomic approaches provide additional value as an important basis for conservation decisions^14,15^.

**Figure 1:**
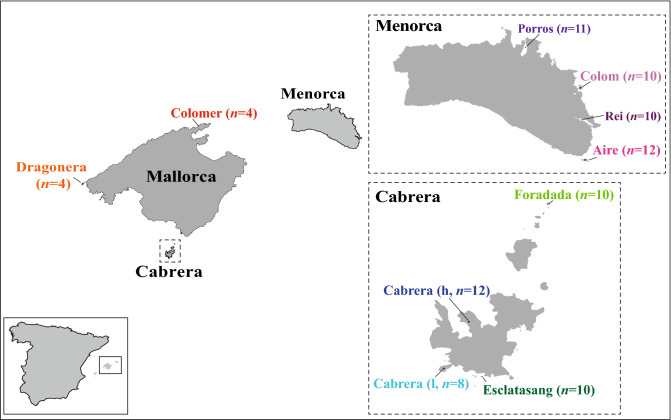
Locations of each coastal island and islet in Mallorca, Menorca and Cabrera and number (n) of samples used in this study. *h* harbour, *l* lighthouse. Figure source: Wikimedia commons.

The Balearic lizard, *Podarcis lilfordi*, as an insular endemism inhabiting a large group of coastal islands and islets of Mallorca and Menorca (Balearic Islands, Spain), provides a suitable system for studying selection and genetic drift as mechanisms of evolution. *Podarcis lilfordi* likely became extinct from the main islands of Mallorca and Menorca during the Holocene (~ 2000 years ago), presumably as a consequence of the introduction of foreign terrestrial predators by humans who arrived 2000–3000 years prior to this^16,17^. Small populations managed to survive on the coastal islands and islets situated around Menorca and Mallorca, as well as the uninhabited Cabrera archipelago (Fig. 1). The sizes of these populations varies considerably, ranging from fewer than 100 individuals, to over 100,000 individuals^18^.

Previous phylogeographical analyses using mitochondrial DNA (mtDNA) have indicated that *P. lilfordi* separated from the Ibizan lizard, *Podarcis pityusensis*, when the Mediterranean refilled at the end of the Messinian Salinity Crisis (~ 5.3 Ma ago). Subsequently, the *P. lilfordi* populations of the islands of Menorca began to diverge from the populations of the islands of Mallorca at the beginning of the Quarternary period, ~ 2.6 Ma ago^19–21^. Despite subsequent glacial events causing sea-level fluctuations^22^, no evidence of historical gene flow or migration can be detected between present-day Mallorcan (including the Cabrera archipelago), and Menorcan populations^20^. Within Mallorca, the earliest split (~ 2 Ma ago) separates the populations of the islands of Western Mallorca from the other populations. The next split within the latter group occurred 1.2 Ma ago and separates northern, southern Mallorcan and northern Cabrera populations from other Cabrera populations. Splits within the latter Cabrera populations are also quite old, with the first estimated at 0.8 Ma^19^. Changes in sea level during the Quarternary were apparently insufficient to reconnect the main islands (Mallorca, Menorca and Cabrera) but would have allowed connections between islets and islands within groups^20^. It is particularly interesting the phylogenetic position of the Colomer Island, an isolated population in northern Mallorca with a steep orography and almost inaccessible nature, that make introductions extremely unlikely. Its closer relationship with populations from the south of Mallorca and Cabrera archipelago seems more probable to be explained by the recent extinction of populations that once inhabited the main island of Mallorca.

The extensive genetic, morphological, ecological and behavioural differences between *P. lilfordi* populations have led to the proposal that they should each be recognized as Evolutionarily Significant Units (ESUs). The range of this species is restricted to a limited geographical area within the Western Mediterranean basin, across which climatic and altitude characteristics vary only slightly^23^. Nonetheless, other environmental traits, such as food availability, habitat structure, orography, predation pressure, the presence of potential competitors and human pressure or some parameter correlated with it, show substantial differences across populations. Here, we aimed to reveal whether these aspects of the environment had led to population divergence.

These well-known populations provide us with a rare opportunity to obtain insight on the effect of short-term environmental changes, most of them driven by humans, in adaptive traits of individuals from a common origin, but now living in different environmental conditions. There are several examples of rapid evolution of species, quickly responding to new selective pressures as human pressure^24,25^. In addition, it is clear that most of the selective pressures associated with humans can be extremely strong and microevolutionary changes can occur on time frames comparable to human disturbance and anthropogenic changes. Such knowledge is crucial to the conservation of biodiversity^26^.

We used double digest restriction-site associated DNA sequencing (ddRADseq), to obtain single-nucleotide polymorphims (SNP) data from across the genome^27–29^. This enabled us to reexamine the population history of *P. lilfordi,* previously described using mtDNA^20^, and explore the roles of genetic drift and divergent selection in shaping genome diversity among these endangered populations.

## Results

A total of 6.8 billion paired-end reads of 101 bp length were generated from the 91 individuals. Following application of *denovo_map.pl* and described filtering steps, 288,286 SNPs were called from 80,091 ddRAD contigs, with a mean coverage of 28.6 per site. The first SNP for each locus was retained leaving 72,846 SNPs for analysis (this number is fewer than the number of loci due to removal of SNPs present in only 20% of individuals).

### Population structure

Nucleotide diversity ranged between 0.120 (Porros islets) and 0.182 (Cabrera harbour). Foradada, Esclatasang and Porros presented the highest number of private alleles (746, 475, and 945, respectively) indicating considerable genetic divergence, with little or no gene flow between them and the other populations, probably due to their strong geographical isolation. In general, inbreeding coefficients (*F*_IS_) were low (less than 10%) (Supplementary Table 1). Patterns of divergence based on *F*_ST_ distance analysis were highly congruent with previous results, with the populations of the islands of Menorca showing a clear differentiation with respect to the populations of the islands of Mallorca together with Cabrera populations (Supplementary Figure 1). Using all 72,846 SNPs, the greatest divergence was between Porros islet (Menorca) and all other populations from Mallorca and Cabrera and between the two Cabrera islets (Foradada and Esclatasang) and Menorcan populations. Lowest divergence was found between the two locations within Cabrera island (harbour and lighthouse), between the populations of the islands of Mallorca (Dragonera and Colomer) and Cabrera main island, and among all Menorcan islands (with the exception of Porros). The divergent position of Porros, Foradada and Esclatasang was less pronounced when only outlier SNPs (1,355 SNPs) were considered, while Mallorca populations were more divergent with respect to Cabrera populations (Supplementary Figure 1).

**Figure 2:**
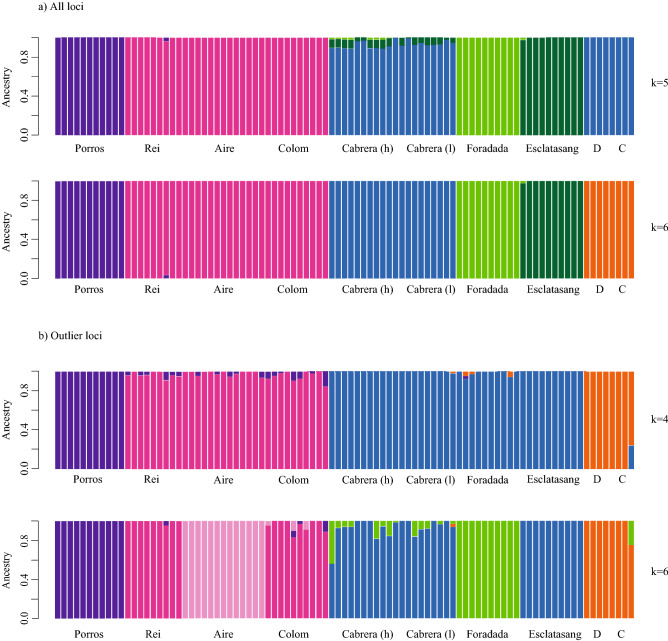
Admixture analysis results using all SNPs dataset (**a**) at *K* = 5 and *K* = 6, and only outlier loci (**b**) at *K* = 4 and *K* = 6. *D* Dragonera, *C* Colomer.

The best-supported values of *K* in the Admixture analysis were *K* = 5 (CV = 0.372) or *K* = 6 (CV = 0.388) for the first single SNPs dataset. The divergent positions of Porros, Foradada and Esclatasang islets was corroborated by these results; Dragonera and Colomer grouped with Cabrera main island with *K* = 5 or formed an independent group with *K* = 6 (Fig. 2). When only outlier SNPs were used, Admixture analyses supported separation into three geographic groups (Menorca, Mallorca and Cabrera), with the exception of Porros islet, when *K* was set to four (CV = 0.288). When *K* = 6 (CV = 0.294), Porros, Aire and Foradada were revealed as independent groups (Fig. 2).

**Figure 3:**
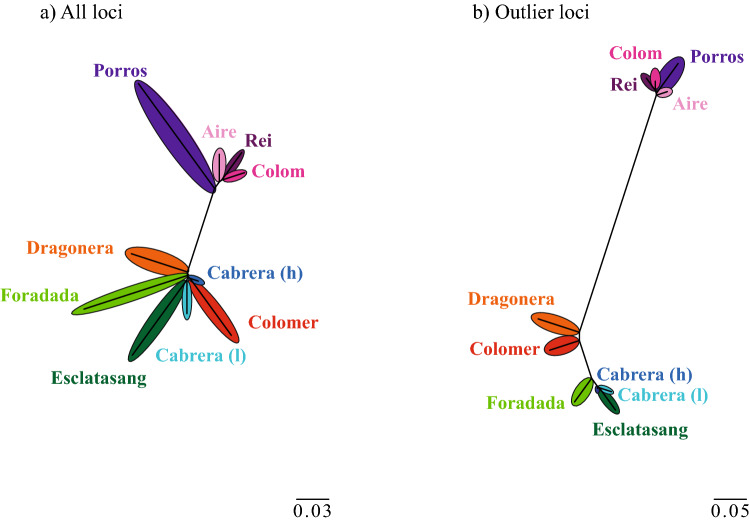
NJ tree based on *F*_ST_ distances based on all SNPs dataset (**a**) and only outlier SNPs (**b**). NJ trees were inferred using Mega 7^60^ and modified with Adobe Illustrator 2020.

Patterns of differentiation observed in the previous analysis match with the population structure obtained with DAPC analyses. The *k*-means clustering algorithm, used prior to DAPC analyses revealed lowest BIC values (637.3) for 10 clusters. Cross-validation showed that use of the first 15 PCs (55.3% of variance) provided higher assignment rates (99.5%) and the lowest root mean squared error (RMSE) (0.016), justifying the use of this subset of PCs in the analysis. The first PC (51.2% of variance) separated all populations into two major groups: Menorcan populations and all the remaining populations from Mallorca and Cabrera. All lizard populations were grouped by island (Cabrera main island, Dragonera, Porros, Aire, Foradada, Esclatasang and Colomer), except for Rei and Colom islets in Menorca that grouped together. Ten clusters were also favored when analyses were carried out using only SNPs that were candidates for selection, and variance was best explained by 25 PCs (90.2% of variance). In this case, the first PC (91.4%) reinforced the clear separation between Menorca islands and Mallorca islands and Cabrera populations. The populations grouped geographically (Menorca, Mallorca and Cabrera), except for Porros islet which continued showing a divergent position (Supplementary Figure 2). NJ tree based on *F*_ST_ distances (Fig. 3) confirmed the results found using the admixture analysis.

**Figure 4:**
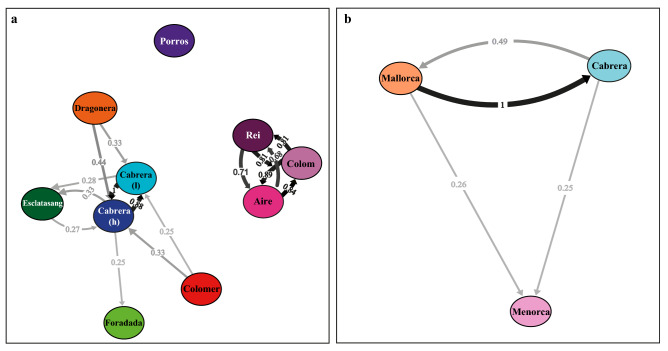
Migration networks for the *Podarcis lilfordi* based on all SNPs (72,846) among the 10 populations (**a**) and between the three main islands (Menorca, Mallorca, Cabrera) (**b**), obtained with the Nei’s *G*_ST_ estimate using divMigrate. Only migration rates ≥ 0.25 are indicated, circles represent the localities, and arrows indicate the direction of migration.

As expected, positive association had been obtained between N and N_e_, and between N and nucleotide diversity (pi) and N_e_ and pi. Negative correlations had been achieved between mean *F*_ST_ and N, but not with N_e_ (data not shown). Migration rates (estimated by divMigrate) did not show gene flow between Menorca islands and Mallorca islands and Cabrera populations (Fig. 4a). The highest migration rates were observed between Aire, Colom and Rei islands in Menorca (0.68–0.89) and between the two localities situated in Cabrera main island (harbour and lighthouse) (0.88–1.00). These migration rates are almost symmetrical. The population from the smallest islet (Porros) did not showed gene flow even with other proximate populations. Directional migration from the populations of the islands of Mallorca (Dragonera and Colomer) to Cabrera archipelago was also observed (0.25–0.44). The Fig. 4b, showed an asymmetric and high migration rate from Mallorca islands to Cabrera archipelago, and low values between Mallorca islands/Cabrera and populations of the islands of Menorca.

### Candidate regions under selection

A total of 1,355 candidate sites for selection from 72,846 RAD tags were determined by BayeScan under a prior of 1:100 for selected:neutral sites. This increased to 2,884 sites when a ratio of 1:10 was used, and decreased to 732 sites when the prior ratio was 1:1000. Comparison of prior and posterior proportions suggests a true ratio between 1:10 and 1:100 and so our use of a 1:100 prior provides quite conservative results. After filtering, a total of 141 of the 184 RAD sites that contained outlying SNPs produced hits on BLASTn and hits with < 30% query coverage were discarded (Table 1).

**Table 1:** Gene ID, definition, Kegg pathway, GO-molecular function and GO-biological process found in *Podarcis* or *Anolis* annotated genomes of the 1,355 outliers SNPs obtained by BayeScan analysis and the posterior filters. References of studies related with specific biological functions are included.

Gene	Definition	Kegg pathway	GO-molecular function	GO-biological process	References
ACACB	Acetyl-CoA carboxylase 2	Fatty acid biosynthesis, pyruvate metabolism, propanoate metabolism, metabolic pathways, insulin signalling pathway, adipocytokine signalling pathway	Acetyl-CoA carboxylase activity, ATP binding, identical protein binding, metal ion binding	Acetyl-CoA metabolic process, fatty acid biosynthesis process, malonyl-CoA biosynthetic process, protein homotetramerization	Lipid metabolism, hibernation^69^
ACSBG1	Acyl-CoA synthetase bubblegum family member 1	Fatty acid biosynthesis, fatty acid degradation, metabolic pathways, fatty acid metabolism, PPAR signalling pathway, adipocytokine signalling pathway	CoA-ligase activity, long-chain fatty acid-CoA ligase activity, very long-chain fatty acid-CoA ligase activity	Long-chain fatty acid biosynthesis process, response to glucocorticoid, very long-chain fatty acid metabolic process	Lipid metabolism, hibernation^70^
ADAM2	ADAM metallopeptidase domain 2		Disintegrins and metallopeptidase activity, metal ion binding, metalloendopeptidase activity, toxin activity	Integrin-mediated signalling pathway	Fertility^71^
ADAM9	ADAM metallopeptidase domain 9		Disintegrins and metallopeptidase activity, collagen binding, metal ion binding, toxin activity Collagen, integrin, laminin. Metal ion and SH3 domain binding, metalloendopeptidase activity, toxin activity	Activation of MAPKK activity, cell–cell adhesion mediated by integrin, cell–matrix adhesion, cellular response to lipopolysaccharide, keratinocyte differentiation, membrane protein ectodomain proteolysis, monocyte activation, positive regulation of cell adhesion mediated by integrin, keratinocyte migration, macrophage fusion and protein secretion, response to calcium ion, hydrogen peroxide, manganese ion, tumor necrosis factor, transforming growth factor beta receptor signalling pathway	Fertility, tail regeneration^71,72^
ADAMTS17	ADAM metallopeptidase with thrombospondin type 1 motif 17		Metal ion binding and metallopeptidase activity	Extracellular matrix organization	^73^
ADCY1	Adenylate cyclase 1	Purine metabolism, metabolic pathways, calcium signalling pathway, oocyte meiosis, adrenergic signalling in cardiomyocytes, vascular smooth muscle contraction, apelin signalling pathway, gap junction, GnRH signalling pathway, progesterone-mediated oocyte maturation, melanogenesis	Adenylate cyclase activity, ATP binding, metal ion binding	Adenylate cyclase-activating G protein-coupled receptor signalling pathway, axonogenesis, cAMP biosynthetic process, long-term memory, neuroinflammatory response, positive regulation of CREB transcription factor activity and long-term synaptic potentiation, reulation of circadian rhythm and synaptic vesicle exocytosis	Circadian rhythm^74^
ADCY2	Adenylate cyclase 2	Purine metabolism, metabolic pathways, calcium signalling pathway, oocyte meiosis, adrenergic signalling in cardiomyocytes, vascular smooth muscle contraction, apelin signalling pathway, gap junction, GnRH signalling pathway, progesterone-mediated oocyte maturation, melanogenesis	Adenlylate cyclase activity, ATP binding, metal ion binding	Adenylate cyclase-activating G protein-coupled receptor signalling pathway, axonogenesis, cAMP biosynthetic process	^73^
ANK1	Ankyrin 1		ATPase binding, cytoskeletal anchor activity, ion channel binding, protein phosphatase binding, spectrin binding	Endoplasmic reticulum to Golgi vesicle-mediated transport, protein localization to plasma membrane	Transcriptional factors, cell regulators, cytoskeletal, ion transporters and signal transducers^75^
ANKRD13A	Ankyrin repeat domain 13A				
CACNA1G	Calcium voltage-gated channel subunit alpha1 G	MAPK and calcium signalling pathway	Voltage-gated calcium and sodium channel activity, scaffold protein binding, cation channel activity	Calcium ion import, cardiac muscle cell action potential involved in contraction, chemical synaptic transmission, membrane depolarization during action potential, neuronal action potential, positive regulation of calcium ion-dependent exocytosis, regulation of atrial cardiac muscle cell membrane depolarization, regulation of heart rate by cardiac conduction, regulation of ion transmembrane transport, response to nickel cation	Sperm storage^76^
CAMK1D	Calcium/calmodulin dependent protein kinase 1D		Calcium signalling pathway. ATP binding, calmodulin binding, calmodulin-dependent protein kinase activity, protein serine/threonine kinase activity	Peptidyl-serine phosphorylation, negative regulation of apoptotic process, positive regulation of apoptotic process, CREB transcription factor activity, neuron projection development, neutrophil chemotaxis, phagocytosis and respiratory burst, regulation of dendrite development	^73^
CNKSR2	Connector enhancer of kinase suppressor of Ras 2		Protein kinase binding	Intracellular signal transduction, regulation of signal transduction	^73^
COL5A3	Collagen alpha-1(XI) chain				
COLGALT1	Collagen beta(1-O)galactosyltransferase 1	Lysine degradation, O-glycan biosynthesis, metabolic pathways	Procollagen galactosyltransferase activity	Positive regulation of collagen fibril organization	Skin development^77^
FGFR1	Fibroblast growth factor receptor 1	MAPK and calcium signalling pathway, adherens junction, regulation of actin cytoskeleton	ATP binding, fibroblast growth factor-activated receptor activity	Positive regulation of cell population proliferation	Tail regeneration^78,79^
GPC1	Glypican 1		Copper ion binding, fibroblast growth factor binding, laminin binding	Cell migration, heparan sulfate proteoglycan catabolic process, negative regulation of fibroblast growth factor receptor signalling pathway, positive regulation of skeletal muscle cell differentiation, regulation of protein localization to membrane	^73^
GPC4	Glypican 4		Wnt signalling pathway	Cell migration, regulation of neurotransmitter receptor localization to postsynaptic specialization membrane, regulation of presynapse assembly, regulation of protein localization to membrane, regulation of signal transduction, synaptic membrane adhesion, Wnt signalling pathway	Adipocyte differentiation^80^
HS6ST2	Heparan-sulfate 6-O-sulfotransferase 2	Glycosaminoglycan biosynthesis—heparan sulfate/heparin	Sulfotransferase activity	Cell proliferation and differentiation	Cell proliferation^81^
ITPR2	Inositol 1,4,5-trisphosphate receptor type 2 isoform X1	Calcium signaling pathway, phosphatidylinositol signalling system, oocyte meiosis, apoptosis, cellular senescence, vascular smooth muscle contraction, apelin signalling pathway, Gap junction, NOD-like receptor signalling pathway, C-type lectin receptor signalling pathway, GnRH signalling pathway	Calcium ion binding, ion channel binding, phosphatidylinositol binding, scaffold protein binding	Cellular response to cAMP and ethanol, release of sequestered calcium ion into cytosol, response to hypoxia	Egg shell quality, muscle contraction, response to hypoxia^82–84^
MAP2	Microtubule associated protein 2		Dystroglycan and microtubule binding	Axonogenesis, cellular response to organic substance, central nervous system neuron development, dendrite morphogenesis, establishment of cell polarity, microtubule bundle formation, microtubule cytoskeleton organization, negative regulation of axon extension, neuron projection development, regulation of cellular protein localization	Neuronal development^85^
MAP7D3	MAP7 domain-containing protein 3 isoform X1			Microtubule cytoskeleton organization	Sex determination^86^
MYO18B	Myosin-XVIIIb		Actin and ATP binding, motor activity		^73^
MYO7B	Myosin VIIb		Actin-dependent ATPase activity, actin filament binding, ATP binding, microfilament motor activity	Actin filament organization, brush border assembly, sensory organ development, sensory perception of sound, vesicle transport along actin filament	^73^
OLFM2	Olfactomedin 2			Positive regulation of smooth muscle cell differentiation, protein secretion	^73^
PBX3	Pre-B-cell leukemia transcription factor 3		DNA binding, DNA-binding transcription factor activity, RNA polymerase II-specific	Animal organ morphogenesis, brain development, embryonic organ development, eye development, neuron development, regulation of transcription by RNA polymerase II	Embryonic development^87^
PCDH17	Protocadherin 17		Calcium ion binding	Adult behaviour, cell adhesion, homophilic cell adhesion via plasma membrane adhesion molecules, negative regulation of synaptic transmission, presynaptic active zone assembly, regulation of synaptic vesicle clustering	^73^
PCDH7	Protocadherin 7		Calcium ion binding	Cell adhesion, homophilic cell adhesion via plasma membrane adhesion molecules	^73^
TACC1	Transforming acidic coiled-coil containing protein 1		Estrogen receptor binding, glucocorticoid receptor binding, peroxisome proliferator activated receptor binding, retinoid X receptor binding, thyroid hormone receptor binding	Cell population proliferation, microtubule cytoskeleton organization, mitotic spindle organization, positive regulation of nuclear receptor transcription coactivator activity	^73^
WNT10A	Protein Wnt-10a	mTOR and Wnt signalling pathway, melanogenesis	Signaling receptor binding	Multicellular organism development, Wnt signalling pathway	Tail regeneration, epidermis morphogenesis^88^
ZNF516	Zinc finger protein 516		Activating transcription factor binding, DNA-binding transcription factor activity, RNA polymerase II-specific, RNA polymerase II cis-regulatory region sequence-specific DNA binding	Adipose tissue development, brown fat cell differentiation, positive regulation of cold-induced thermogenesis and transcription, response to cold	Thermogenesis^89^
ZNF711	Zinc finger protein 711		DNA binding, metal ion binding	Regulation of transcription	^73^

### Environmental association analysis

**Figure 5:**
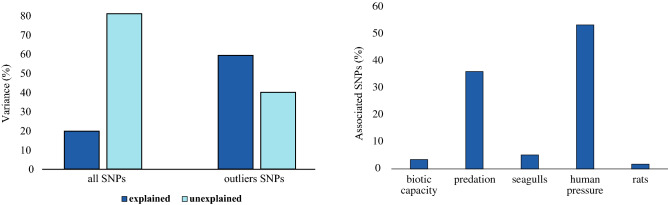
Variation explained by ecological variables computed in the RDA analysis based on all SNPs (72,846) and outlier SNPs only (1355) is indicated on the left graph. The percentage of associated SNPs for the retained variables after RDA analysis based on SNPs under selection is indicated on the right graph.

The RDA analysis that used all SNPs indicated that the variation explained by the environmental variables (20.1%) was lower than the unexplained variance (79.9%) (Fig. 5). However, when the analysis was based on only outlier SNPs (1,355), environmental variables explained most of the variation (60.4%). The low explanatory power obtained with all SNPs is not surprising given that we expect that most of the SNPs in our global dataset to be neutral and not associated with environmental predictors. A total of 58 loci with associations with environmental variables were detected, most of which were related to human pressure (53.5%) and predation (36.2%). Some of these associated SNPs have been found to be related to locomotory and feeding behavior (NEGR1, GRM1), perception of pain (GRM1), lipid metabolism (GDPD2) or ion transport (FHL1, FTH1, SLC9A6), microtubule formation (CLIP1), myoblast differentiation (MBNL3), embryonic development (INTS6L), pH regulation (SLC9A6), toxin transport (DNAJC17), cell adhesion (ESAM, NEGR1), hormone regulation (TG, NCOA1), brain development and cognition (SHROOM4).

## Discussion

The RADseq methodology has been applied in other studies of squamate (lizards and snakes), increasing understanding of the processes related to genetic divergence and the identification of genomic regions of interest. The total number of SNPs obtained in this study (288,286) agree with the SNP density found in other RADseq studies of reptiles, with relatively high levels of diversity detected^30,31^. Population structure analysis revealed a clear genetic structure among all the populations of *P. lilfordi*, independent of whether we used SNPs from all RAD tags or just candidates for selection^32^. Major genetic structuring mirrors that found using mtDNA, with high levels of divergence between Menorca islands and Mallorca islands/Cabrera populations^19,20^. However, analyses of outlier SNPs revealed greater similarity between the northern Mallorca Colomer population and other Mallorca islands (Dragonera), which differs from the pattern found in mtDNA^20^. These results together with the high migration rate detected between Mallorca islands and the Cabrera archipelago populations supports a previous proposal^20^ that the Colomer Island could be home of a relict population representative of the early population that once colonized Mallorca Island.

The populations with the smallest population sizes (Porros, Foradada and Esclatasang islets) were most divergent with highest *F*_ST_ values and the greatest number of private alleles relative to other populations, which supports previous findings^30,33^ and is suggestive of genetic drift. Long-term isolation and small population size should lead to decreased genetic diversity and increased inbreeding coffecients^34–37^. While nucleotide diversity was low^38^, inbreeding values were under 10%^39,40^ which is not indicative of an inbreeding effect.

It is worth highlighting evidence of adaptive divergence among lizard populations based on *F*_ST_ outlier tests. Almost 2% of total SNPs were candidates for selection. These loci were related to several functions with direct survival value such as tail regeneration, reproduction, lipid metabolism and circadian rhythm. Nonetheless, the still incomplete annotation of the available *Podarcis* genome makes necessary a more in-depth analysis to elucidate the molecular mechanisms of adaptation in this genus. Other studies of lizards have revealed links between genetic variation of candidate genes and geographical distributions, patterns of colonization and/or landscapes gradients^13,41–43^.

We show that environmental variables appear to be an important driver of divergence between lizard populations after taking into account the effect of historical divergence. The RDA analysis revealed most SNPs that were influenced by the environment were associated with levels of predation and human pressure. These SNPs were involved in diverse functions most notably with feeding and locomotory behavior. The explanatory power of the remaining environmental predictors, such as the biotic capacity of islands, the presence of rats, or the existence of breeding colonies of gulls, is negligible. Some behavioral and physiological differences between populations can be related to differences in predation and human pressures, as in the case of escape behavior in lizard populations with or without terrestrial predators. For example, predation pressure has previously been shown to influence flight initiation distance, distance fled, or hiding time in Balearic lizard populations^44–47^.

Predation has traditionally been identified as a major selective factor shaping the morphological and demographic evolution of animal species^48^. Unlike many terrestrial vertebrates that have evolved in the presence of these selection pressures over millions of years, *P. lilfordi* has evolved for ~ 5.3 Ma in a pristine environment, free from terrestrial predators^16^. The subsequent arrival of humans ~ 5000 years ago caused a major change as allochthonous predators were introduced. Hence there is a strong association between indices of human pressure and predation pressure as a result of this Holocenic arrival^16,17,49^.

It is interesting that this selection has had a strong and detectable effect on the genomic structure of these populations in a relatively short time. This has been described in a few other studies^50–52^. However, to our knowledge, this is the first case where predator and human pressures have been functionally linked with possible selection on loci involved in physiological functions that are directly involved with locomotor and escape behaviors. Same human-driven factors are often responsible of rapid adaptation and current extinction crisis^53^. This fact implies that the study of rapid adaptation to novel environment changes, especially those related with humans, has an inmediate relevance to conservation biology. For this reason, the study of adaptive evolution need to be incorporate into conservation strategies of insular terrestrial vertebrates populations and specifically in the Balearic lizard. In this way, Ashley et al.^25^ proposed the promotion of an evolutionary enlightened management in which conservation decisions need to take into account the evolutionary effects of anthropogenic changes.

Overall, our results reveal that both evolutionary processes, associated with isolation and small population size, and selective factors, related to environmental patterns (specifically human pressure and level of predation) have played a role in shaping divergence between Balearic lizard populations.

## Methods

### Sample collection, DNA extraction, library preparation, and sequencing

Tissue samples were collected from 94 lizards (*P. lilfordi*) from 10 different sampling locations across the Balearic archipelago (Fig. 1 and Table 2). Populations were selected to cover a diverse range of substrates, orographies, plant cover, presence of terrestrial predator and human pressure, as well as different population sizes and different mtDNA clades (Table 2). Total genomic DNA was extracted from each tissue sample using DNeasy Blood and Tissue Kit (Qiagen, Hilden, Germany) following the manufacturer’s standard protocol with a specific RNase copurification step. DNA was quantified using the Thermo Fisher Scientific Qubit 3.0 Fluorometer (ThermoFisher Scientific) and quality evaluated using agarose gel and Nanovue Plus Spectrophotometer (GE Healthcare, UK Limited). Paired-end ddRADseq libraries were prepared and sequenced by Floragenex (Eugene, Oregon, USA), following Peterson et al.^28^ and Truong et al.^54^ protocols. Full details are provided in Supplementary Methods.

**Table 2:** Characteristics and environmental variables of the studied populations. *n* number of samples used for every population, *S* island surface area in hectares, *predation indexes* absence of terrestrial predators = 0; one occasional predators in the island = 1; one widespread predator was or is present in the island = 2; two frequent predators present in the island = 3, *human pressure* uninhabited island and very difficult access = 0; sporadic human presence and easy access = 1; regular human presence and easy access = 2; previous permanent human presence with constructions but with an actual protection = 3; present and past human presence = 4.

Population	n	Population size (N)	S (ha)	Biotic capacity	Vascular plants	Predation	Human pressure	Rats	Seagulls
**Menorca**									
Aire	12	77,500	29.8	6.10	94	0	2	No	Yes
Colom	10	58,107	51.14	7.62	267	1	3	Yes	Yes
Porros	10	54	0.05	−2.66	32	0	1	No	No
Rei	10	1845	4.08	4.08	204	2	4	Yes	No
**Cabrera**									
Cabrera (harbour)	12	534,888	1137.24	12.18	486	3	3	Yes	Yes
Cabrera (lighthouse)	10	5171	10.6	7.16	486	3	2	Yes	Yes
Esclatasang	11	714	0.42	2.69	23	0	0	No	Yes
Foradada	10	1356	1.61	3.77	19	0	1	No	Yes
**Mallorca**									
Dragonera	4	132,875	267.81	11.29	300	0	2	Yes	Yes
Colomer	4	10,017	3.05	5.74	8	0	0	No	Yes

### Data processing and variant calling

Stacks v2.4^55^ pipelines were used to process the sequence reads and call SNPs for each individual. First, a demultiplexing and quality filtering step was carried out using *process_radtags* with the default parameters. Clean reads were used to perform a de novo RAD assembly using the *denovo_map.pl* pipeline. The percentage of missing genotypes for each individual was calculated using the *-missing-indv* in VCFtools v0.1.15^56^ and three individuals with more than 79% of missing data were removed. SNPs present in RAD tags found in at least 80% (R) of individuals (Supplementary Figure 3) and with a minimum allele frequency (MAF) of 0.05 were selected and exported into a VCF file using *populations*. One single SNP per RAD tag was called using *populations* to reduce the effects of linkage disequilibrium. See Supplementary Methods.

### Population structure

Several analyses were used to characterize population structure of island lizard populations based on all RAD-tag information (single SNP selected from each tag, referred to as the all-SNP dataset: VCF file in Appendix S1) and using only outlier SNPs (see later for identification of outliers: VCF file in Appendix S2). First, two different programs, Stacks v4.2^55^ and *hierfstat* R package^57^, were used to estimate levels of genetic variability among different lizard populations. Second, population structure was examined with Admixture v1.3.0 program^58^ based on both datasets, for K = 2 to K = 10 co-ancestry clusters. Third, patterns of genetic divergence on both datasets were analyzed using two approaches. Discriminant Analysis of Principal Components (DAPC) was performed using the R package *adegenet*^59^ to obtain an overall representation of the divergence between populations and Neighbor-Joining (NJ) trees were inferred using Mega 7^60^ based on pairwise *F*_ST_ distances.

Effective population size (N_e_) for each population has been estimated with the software NeEstimator v2.0.1^61^ using the molecular coancestry method. Linear regression analyses between N and N_e_, pi and N, pi and N_e_, and *F*_ST_ with N and with N_e_, was performed with Pearson correlation. To investigate migration rates between each locality and between each island (Mallorca, Menorca and Cabrera), migration networks were generated usind *divMigrate* function^62^ in the R package *diveRsity*^63^ based on *G*_ST_ genetic distance^64^ with 1000 bootstrap repetitions and a filter threshold of 0.25. More information is provided in the Supplementary Methods.

### Test of selection and environmental association analysis

Tests of selection was carried out to explore the role of divergent selection using BayeScan^65^. This program identifies candidate loci under selection using an *F*_ST_ outlier approach across all sampled populations. The BayeScan algorithm is based on an island model in which subpopulations differ from a common migrant pool. Thus, a departure from neutrality is identified at a SNP when the overall genome divergence between different subpopulations is insufficient to explain its diversity across these subpopulations.

Genome-environment association (GEA) is an important tool for the examination of local adaptation to heterogeneous landscapes^66,67^. Climatic variables were not used as environmental predictors because the Balearic lizard inhabits a reduced geographical range with minimal climatic variation^23^. Six environmental traits were considered: biotic capacity, number of vascular plants species, predation pressure, human pressure, and presence/absence of rats and gulls. All of these traits are related to natural resources on the islands and factors that potentially affect the lizards’ survival and were known to show clear differences among the populations studied. Partial redundancy analysis (RDA) was used as a GEA method to identify adaptive loci based on associations between genetic data and environmental predictors^68^. See Supplementary Methods.

### Ethical statement

All tail tips samples used in this study were obtained in accordance with Ethical Guidelines of the Universities of Balearic Islands and Salamanca, particularly, following the Bioethics Committee Guidelines of the University of Salamanca. The Ethical Committee from the University of Salamanca publishes general Guidelines concerning the experimental protocols with laboratory animals. These general Guidelines for laboratory animals can be read in http://www.usal.es. According to these Guidelines, only the requirements applicable to our study were implemented simply because we did not perform any experiment with lizards in captivity. Field protocols for the capture, handling and release of lizards (which was done at the site of capture a few minutes after the sampling of tail tips) were approved by the competent authority: the Nature Conservation Agency (Conselleria de Medi Ambient) of the Government of Balearic Island (permits: CEP 02/2018 and CEP 10/2016 to V.P.-M. and A. P.-C.).

## Supplementary Information

Supplementary Appendix S1.

Supplementary Appendix S2.

Supplementary Figure 1.

Supplementary Figure 2.

Supplementary Figure 3.

Supplementary Figure Legends.

Supplementary Information.

## Data Availability

Individual raw sequences are available at the Sequence Read Archive (SRA) (BioProject ID: PRJNA645796). The VCF files with first single SNPs and only with outlier SNPs putatively under selection are found on Appendices S1 and S2.
