# Supplementary material for: Genomic signatures of drift and selection driven by predation and human pressure in an insular lizard
Source: Sci Rep. 2021 Mar 17;11:6136. doi: 10.1038/s41598-021-85591-x (PMC7971075; doi:10.1038/s41598-021-85591-x)
Supplement: Supplementary file 3 — Supplementary Figure 1. [file 41598_2021_85591_MOESM3_ESM.pdf]

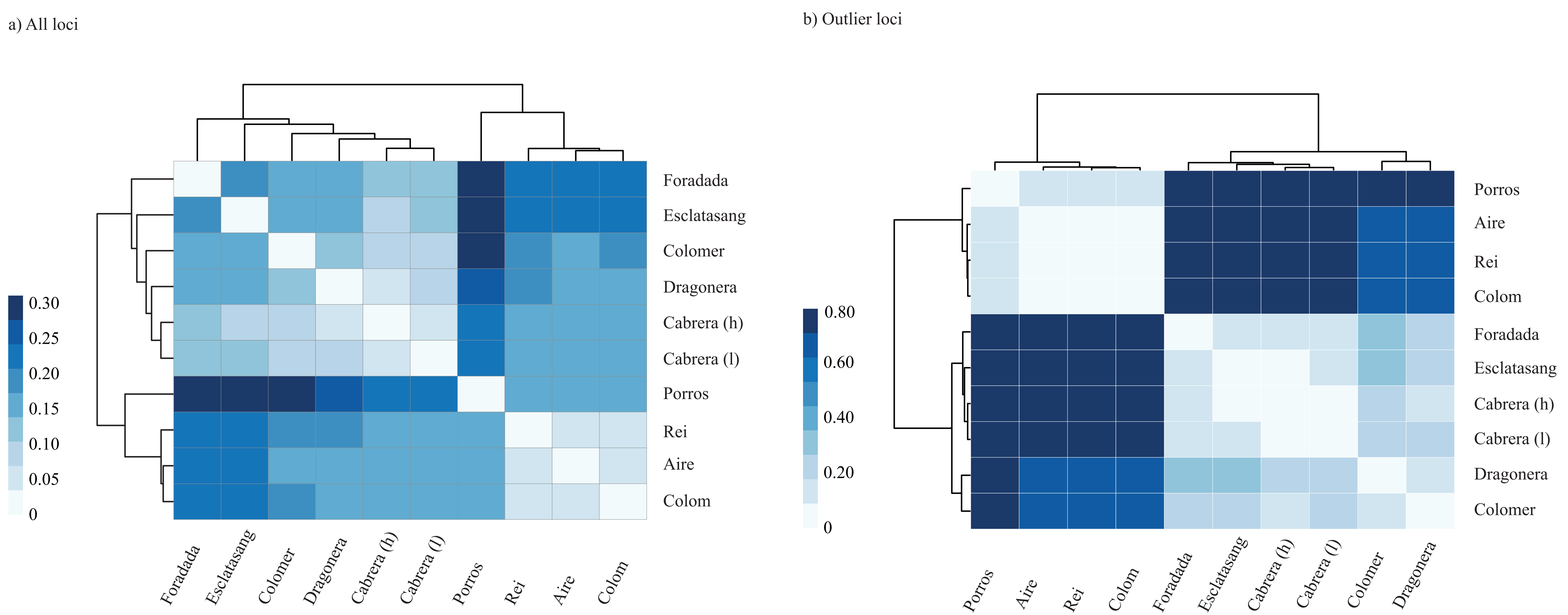

a) All loci
b) Outlier loci
Foradada
Esclatasang
Colomer
Dragonera
Cabrera (h)
Cabrera (l)
Porros
Rei
Aire
Colom
0.30
0.25
0.20
0.15
0.10
0.05
0
0.80
0.60
0.40
0.20
0
