# Supplementary figures and images for: Genomic signatures of drift and selection driven by predation and human pressure in an insular lizard
Source: Sci Rep. 2021 Mar 17;11:6136. doi: 10.1038/s41598-021-85591-x (PMC7971075; doi:10.1038/s41598-021-85591-x)

a) All loci

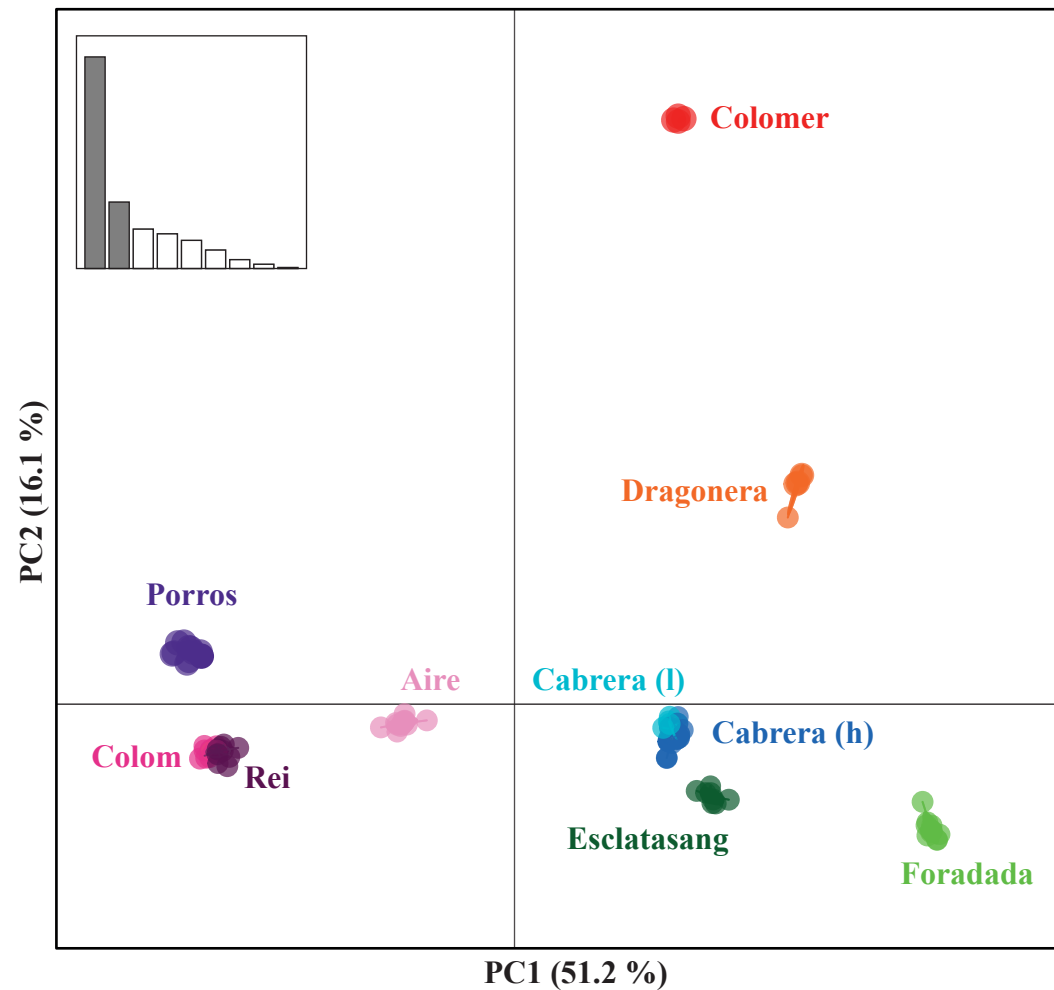

b) Outlier loci

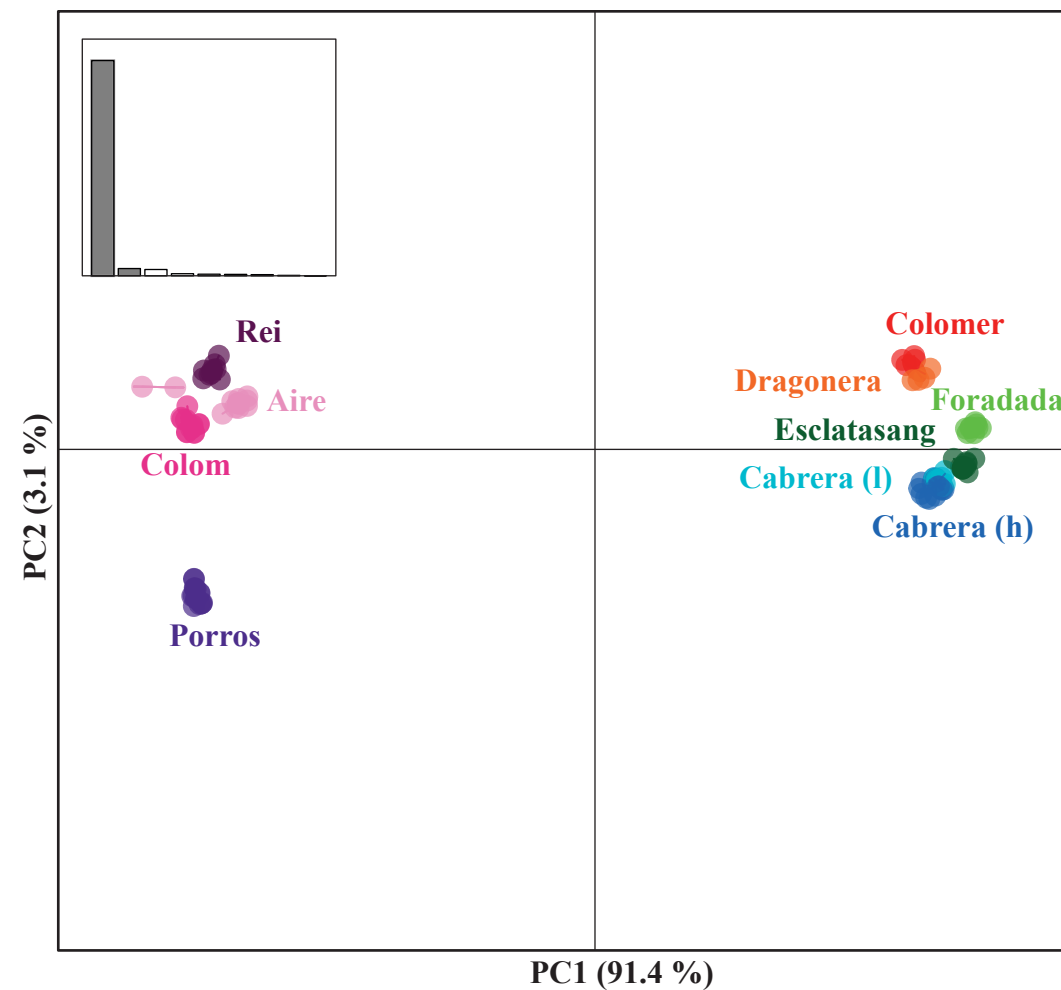

Supplement: Supplementary file 4 — Supplementary Figure 2. [file 41598_2021_85591_MOESM4_ESM.pdf]

### Polymorphic loci

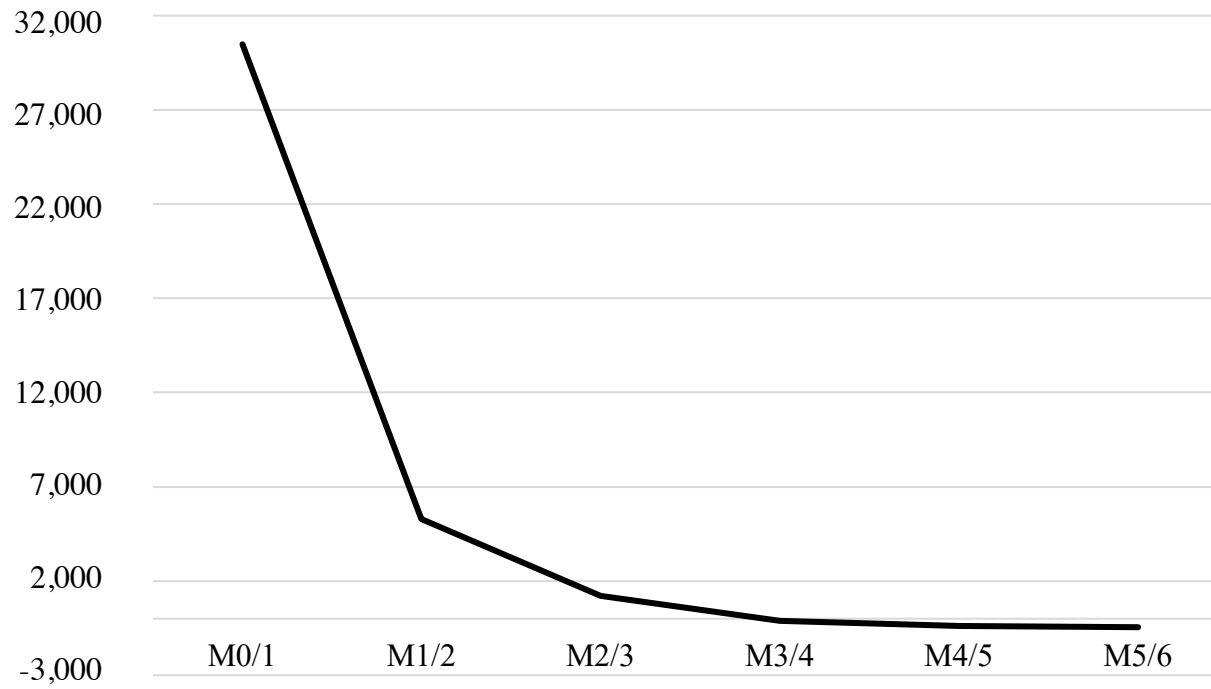

Supplement: Supplementary file 5 — Supplementary Figure 3. [file 41598_2021_85591_MOESM5_ESM.pdf]
