# Supplementary material for: Genomic signatures of drift and selection driven by predation and human pressure in an insular lizard
Source: Sci Rep. 2021 Mar 17;11:6136. doi: 10.1038/s41598-021-85591-x (PMC7971075; doi:10.1038/s41598-021-85591-x)
Supplement: Supplementary file 6 — Supplementary Figure Legends. [file 41598_2021_85591_MOESM6_ESM.docx]

**Supplementary Figures and Appendices legends**

Supplementary Figure 1. Heatmaps based on genetic distances (*F*_ST_ values) among studied populations for first single SNPs (a) and for outlier SNPs (b). Heatmaps were obtained with ggplot2 package in R (<https://cran.r-project.org/web/packages/ggplot2/index.html>).

Supplementary Figure 2. DAPC analysis results with first single SNPs (a) and only outlier loci (b). Abbreviations: h, harbour; l, lighthouse.

Supplementary Figure 3. Plot of the number of new polymorphic loci added for each iteration of M=n for a subsample of our database using r80 method [7].

Appendix S1. The 72,846 first single SNPs in vcf format.

Appendix S2. The 1,355 outlier SNPs in vcf format.
