# Supplementary material for: Genomic signatures of drift and selection driven by predation and human pressure in an insular lizard
Source: Sci Rep. 2021 Mar 17;11:6136. doi: 10.1038/s41598-021-85591-x (PMC7971075; doi:10.1038/s41598-021-85591-x)
Supplement: Supplementary file 7 — Supplementary Information. [file 41598_2021_85591_MOESM7_ESM.docx]

**Genomic signatures of drift and selection driven by predation and human pressure in an insular lizard**

**Supplementary Material**

Supplementary Methods, Supplementary Tables

**Authors:**

Marta Bassitta^1*^, Richard P. Brown^2^, Ana Pérez-Cembranos^3^, Valentín Pérez-Mellado^3^, José A. Castro^1^, Antònia Picornell^1^ and Cori Ramon^1^

^1^Laboratori de Genètica, Departament de Biologia, Universitat de les Illes Balears, Crta. de Valldemossa, km 7.5, 07122, Palma de Mallorca, Illes Balears, Spain

- ^2^ School of Biological & Envrionmental Sciences, Liverpool John Moores University, Liverpool, United Kingdom
- ^3^Departamento de Biología Animal, Universidad de Salamanca, Campus Miguel de Unamuno, Edificio de Farmacia, 37071 Salamanca, Spain
- *Correspondence: [m.bassitta@uib.es](mailto:m.bassitta@uib.es)

### Supplementary Methods

- *Sample collection, DNA extraction, library preparation, and sequencing*
- Lizards were caught by careful noosing and 1 cm of tail tip was removed and stored in DNAgard® or in ethanol 100%. All individuals were realised at sites of capture after sample collection. The number of individuals sampled in each location ranging between 4 and 12. Populations were selected from 43 known *P. lilfordi* extant subspecies [1] to cover the diversity present in the different lizard populations (Table 2). For each individual, 1 µg of genomic DNA, extracted as indicated in Methods, was sent to Floragenex (Eugene, Oregon, USA) for ddRADseq library construction, following the protocols described in [2] and [3], with modifications. Briefly, 100-500 ng total genomic DNA was double digested using PstI (CTGCAG) and MseI (TTAA). After digestion, sequence identifier barcodes and sequence adapters were added, and PCR was performed. A 3’ primer that selects for fragments which have a “TC” in front of the MseI site was used to decrease complexity. Paired-end sequencing with read length of 101 bp was conducted using one lane of the Illumina HiSeq 4000 at the University of Oregon Genomics and Cell Characterization Core Facility.
- *Data processing and variant calling*
- Raw read sequences quality was checked using FastQC v0.10.1 [4] and then processed using the *denovo_map.pl* pipeline in Stacks v2.4 [5]. First, reads were demultiplexed and filtered using *process_radtags* pipeline. Low quality score (Phred score was set to 10), uncalled nucleotides, and unidentifiable barcode or restriction site were discarded. Clean reads were used to perform *de novo* RAD assembly using the *denovo_map.pl* pipeline. RAD tags within individuals were aligned using *ustacks* with parameters *m* (minimum depth of coverage required to form a stack) and *M* (maximum nucleotide mismatch allowed to merge two stacks) set to three. RAD tags were combined into a catalogue using *cstacks* allowing up to three mismatches between loci (n). Matches of individual RAD tags to the catalogue were searched using *sstacks*. The percentage of missing genotypes for each individual was calculated using the *--missing-indv* in VCFtools v0.1.15 [6] and three individuals with more than 79% of missing data were removed. SNPs present in RAD tags found in at least 80% (R) of individuals and with a minimum allele frequency (MAF) of 0.05 were selected and exported into a VCF file using *populations*. One single SNP per RAD tag was called using *populations* to reduce the effects of linkage disequilibrium. Parameter selection for *de novo* pipeline was based on the r80 optimization approach [7]. Optimization tests were run on a subset of 10 individuals (one per population). Different values of (M) and (n) (1 to 6) were tested, with (m) fixed as 3 and R as 0.80. The effects of M and n were evaluated on the number of polymorphic loci obtained with *population* in Stacks (Supplementary Figure 3). The SNPs dataset was exported to BayeScan [8] format using PGDSpider v2.1.1.5 [9] and to Admixture v1.3.0 [10] format using PLINK v1.90 [11].
- *Population structure*
- Two different programs were used to obtain indices of population-specific genetic variability based on the entire genome dataset. Stacks v4.2 [5] software was used to calculate the number of private alleles, nucleotide diversity (π), observed (Ho) and expected heterozygosity (He), and inbreeding coefficient (*F*_IS_) for all positions and for variant positions. Genome-wide estimates of divergence (*F*_ST_) were calculated between all populations applying a p-value correction (p<0.001) to exclude insignificant *F*_ST_ measures. The *hierfstat* R package [12] was used to obtain allelic richness (Ar) H_o_, H_e_, *F*_IS_ and *F*_ST_ using the *basic.stats* function and bootstrap confidence intervals (1,000 bootstrap replicates). Confidence intervals for population specific *F*_IS_ and pairwise *F*_ST_ were determined using the *boot.ppfis* and *boot.ppfst* functions, respectively. Pairwise *F*_ST_ following [13] was calculated using the *genet.dist* function for both datasets (first single SNPs and only outlier SNPs). Heatmaps based on *F*_ST_ were created using ggplot2 in R [14].
- Admixture v1.3.0 program [10] was used to estimate population structure based on first single SNPs and only on outlier loci, assuming different numbers of co-ancestry clusters (*K*) ranging from 2 to 10. Cross-validation (CV) was set to 10-fold to compare different number of *K*, in which lower CV value indicates the most likely number of clusters.

Patterns of genetic divergence were analyzed using two different approaches based on the entire dataset and only on SNPs putatively under selection. A Discriminant Analysis of Principal Components (DAPC) was performed using the R package *adegenet* [15] to obtain an overall representation of the divergence between populations. In the first step of this analysis, a Principal Components Analysis (PCA) is applied to examine differentiation between individuals regardless of population origin. In the second step, the Principal Components (PCs) that provide suitable predictive power, as assessed by cross-validation, are input into a Discriminant Function Analysis (DFA). Individuals were grouped by population for the DFA, which maximizes among-group relative to within-group variation. Neighbor-Joining (NJ) trees were inferred using Mega 7 [16] based on pairwise *F*_ST_ distances for both datasets.

- Migration rates were estimated with *divMigrate* package, which method is based on defining a hypothetical pool of migrants for each pair of populations and estimating the genetic differentiation between each of the two populations and the hypothetical pool. The genetic differentiation is used to estimate the relative levels of migration between the two populations. The larger migration values indicate the population that is likely to behave as a source population (hypothetical pool of migrants is genetically more similar to this population), while the smaller of the two estimates indicates the population most likely to behave as a sink [17].

*Candidate regions under selection*

- BayeScan [8] was used to identify candidate loci under selection based on an *F*_ST_ outlier approach. The method implements a reversible jump MCMC approach that can move between a selection model, containing a population-specific component and a locus-specific component, and a model with just a population-specific component (no selection). The posterior probability of selection at a locus is determined by the proportion of MCMC samples that include the model with the locus-specific component. The prior odds specified for the ratio of neutral:selected sites were set at 1:100, reflecting that neutral sites might be around 100 times more probable than sites under selection [18]. This prior can have considerable influence on the number of sites detected, so runs were also carried out with 1:10 and 1:1000 proportions. The MCMC characteristics of the analysis were: 20 pilot runs of 5,000 steps, 5,000 iterations with 50,000 discarded as burnin, and a sampling interval of 10. Outliers were identified from the results using the R code supplied with Bayescan and significance was determined under a false discovery rate of 5%. We searched the Kegg pathway (Kegg Pathway Database), and the GO-molecular functions and GO-biological process using UniProtKB database [19] in *Podarcis* (NCBI:txid42163) or *Anolis* (NCBI:txid28376) genome for outliers with high F_ST_ (> 0.80) and log_10_(PO) equal to 1000 that blasted to genes in the *Podarcis* genome.
- *Environmental predictors*
- Biotic capacity, number of vascular plants species, predation pressure, human pressure, and presence/absence of rats and gulls were considered as environmental variables for the genome-environment association analysis. The environmental variables used in this study were selected because they showed clear differences among the populations studied, such as the presence of potential competitors and predators, as well as orographic and landscape traits that can be considered *proxy* of habitat heterogeneity and resource availability.
- As an indicator of orography and habitat diversity, for each island we recorded its maximum altitude to calculate the so-called index D of “biotic capacity” [20, 21], with the formula: D= log(S x a), where *S* is the surface of the island (in hectares) and *a* is its maximum altitude (in meters). The number of vascular plants was employed as an indicator of resource availability in each population. For this variable, data were obtained from several sources [22, 23, unpublished lists and personal observations]. The remaining environmental predictors were codified as categorical variables, as performed in a previous study [23] (Table 2). Four levels of predation pressure were considered: 0= absence of terrestrial predators; 1= one occasional predator present on the island: there is only one case, Colom Island, where the Ladder snake, *Zamenis scalaris*, an introduced species that very rarely predates on lizards, is present [24]; 2= at least one regular predator on lizards was or is present on the island: this is the case of Rei Island, where domestic cats, *Felis catus* were present for centuries, when the military hospital was active [25]; 3= two regular predators on lizards present on the island, such as Cabrera Island, where two carnivorous mammals are still present: the feral cat, *Felis catus* and the Genet, *Genetta genetta*, both of which are important predators of lizards [1]. The Yellow-legged gull (*Larus michahellis*) hardly ever captures lizards [26] and cannot be considered a lizard predator. Human pressure was codified in four levels: 0= uninhabited island, absence of human visits and/or very difficult landing. This is the case of Colomer Island in Mallorca and Esclatasang islet in Southern Cabrera, both are protected areas with very difficult landing; 1=sporadic human presence and easy landing; 2= regular human presence and easy access; 3= previous permanent human presence with constructions but with an actual protection that mitigates present day human pressure; and 4= present and past human presence. We included two additional variables for the presence of other vertebrate species that can act, potentially, as competitors of lizards for trophic resources: *Rattus rattus* and two breeding gulls, *Larus michahellis* and *Ichtyaetus audouinii* [23 and personal observations].
- Lizard densities were estimated during the spring of 2018, employing the line transect method [27] with the R package *unmarked*. This package fits hierarchical models of animal occurrence and abundance to data collected on species subject to imperfect detection [28]. Then, lizard population sizes were estimated as the total number of lizards present in each population with that density (lizards/hectare) and the surface covered with vascular plants, estimated from the satellite view of Google maps and personal observations.
- *Environmental association analysis*
- RDA is a multivariate constrained ordination method that can be used to explain differences in population structure using a combination between linear regression and principal component analysis [29]. Constrained ordinations explain the relative contribution of the chosen environmental variables in the genetic structure [30]. In this study, RDA was performed using *vegan* R package [31] based on the entire genome data and using the subset of outlier identified by BayeScan.

To estimate the proportion of genetic variation in lizard populations explained by divergent selection (environmental variables), RDA was run including environmental variables (Table 2). RDA analysis was performed imputing missing genotypes on the SNP loci dataset by replacing them with the most common genotype across all individuals. For determination of best model, we applied a forward stepwise selection process with permutation of 999 and *a*=0.01 using *ordiR2step* function, maximizing adjusted R^2^ at every step [31]. To avoid collinearity and redundancy information, retained variables with a variance inflation factor (VIF) over 10 were excluded [29]. Finally, RDA analysis was carried out with five environmental variables (predation, human pressure, biotic capacity and presence of seagulls and rats) to obtain the percentage of associated SNPs related to each variable. The UniProtKB database [19] was used to obtain GO biological process and molecular functions of associated SNPs with the highest percentage.

**References**

1. Pérez‐Cembranos, A. et al. Morphological and genetic diversity of the Balearic lizard, *Podarcis lilfordi* (Günther, 1874): Is it relevant to its conservation? *Divers. Distrib*. 26, 1122-1141 (2020).
2. Peterson, B.K., Weber, J.N., Kay, E.H., Fisher, H.S. & Hoekstra, H.E. Double digest RADseq: an inexpensive method for de novo SNP discovery and genotyping in model and non-model species. *PloS one* 7(5), e37135 (2012).
3. Truong, H.T. et al. Sequence-based Genotyping for Marker Discovery and Co-Dominant Scoring in Germplasm and Populations. *PLoS One* 7(5), e37565 (2012).
4. Andrews, S. FastQC: a quality control tool for high throughput sequence data. Available online at: <http://www.bioinformatics.babraham.ac.uk/projects/fastqc> (2010).
5. Catchen, J., Hohenlohe, P.A., Bassham, S., Amores, A. & Cresko, W.A. Stacks: an analysis tool set for population genomics. *Mol. Ecol.* 22(11), 3124-3140 (2013).
6. Danecek, P. et al. The variant call format and VCFtools. *Bioinformatics* 27(15), 2156-2158 (2011).
7. Paris, J.R., Stevens, J.R. & Catchen, J.M. Lost in parameter space: a road map for Stacks. *Methods Ecol. Evol*. 8(10), 1360-1373 (2017).
8. Foll, M. & Gaggiotti, O. A genome-scan method to identify selected loci appropriate for both dominant and codominant markers: a Bayesian perspective. *Genetics* 180(2), 977-993 (2008).
9. Lischer, H.E. & Excoffier, L. PGDSpider: an automated data conversion tool for connecting population genetics and genomics programs. *Bioinformatics* 28(2), 298-299 (2012).
10. Alexander, D. H., Novembre, J. & Lange, K. Fast model-based estimation of ancestry in unrelated individuals. *Genome Res*. 19(9), 1655-1664 (2009).
11. Chang, C. C. et al. Second-generation PLINK: rising to the challenge of larger and richer datasets. *Gigascience* 4(1), s13742-015 (2015).
12. Goudet, J. & Jombart, T. hierfstat: Estimation and tests of hierarchical F-statistics. R package version 0.5-7. Available from <http://github.com/jgx65/hierfstat> (2015).
13. Weir, B.S. & Cockerham, C.C. Estimating F-statistics for the analysis of population structure. *Evolution* 38(6), 1358-1370 (1984).
14. Wickham, H. & Chan, W. Ggplot. R package version 2.21. Online: https://github. com/tidyverse/ggplot2 (2016).
15. Jombart, T. & Ahmed, I. Adegenet 1.3-1: new tools for the analysis of genome-wide SNP data. *Bioinformatics* 27(21), 3070-3071 (2011).
16. Kumar, S., Stecher, G. & Tamura, K. MEGA7: molecular evolutionary genetics analysis version 7.0 for bigger datasets. *Mol. Biol. Evol.* 33(7), 1870-1874 (2016).
17. Sundqvist, L., Keenan, K., Zackrisson, M., Prodöhl, P., & Kleinhans, D. Directional genetic differentiation and relative migration. *Ecol. Evol*. 6(11), 3461-3475 (2016).
18. Lotterhos, K.E. & Whitlock, M.C. Evaluation of demographic history and neutral parameterization on the performance of FST outlier tests. *Mol. Ecol.* 23(9), 2178-2192 (2014).
19. The UniProt Consortium. UniProt: The universal protein knowledgebase. *Nucleic Acids Res*. 45, D158–D169 (2017).
20. Cheylan, M. Synthèse biogéographique in *Atlas de Répartition des Batraciens et Reptiles de Corse* (ed. Delaguerre, M. & Cheylan, M.) 105-120 (Montpellier, Parc Naturel Régional de Corse/École Pratique des Hautes Études, 1992).
21. Parlanti, C., Lanza, B., Poggesi, M. & Sbordoni, V. Anfibi e Rettili delle isole del Mediterraneo: un test dellípotesi dell’equilibrio insulare. *Bulletin d’Ecologie* 19, 335- 348 (1988).
22. Bibiloni, G., Alomar, G. & Rita, J. XII. Flora vascular dels illots i addicions a la flora vascular de Cabrera gran in *Història Natural de l’Arxipèlag de Cabrera*. (ed. Alcover, J.A., Ballesteros, E. & Fornós, J.J.) 179- 206 (Pama de Mallorca, CSIC & Editorial Moll, 1993).
23. Pérez-Mellado, V. et al. Population density in *Podarcis lilfordi* (Squamata, Lacertidae), a lizard species endemic to small islets in the Balearic Islands (Spain). *Amphibia-Reptilia* 29(1), 49-60 (2008).
24. Pleguezuelos, J.M. Elaphe scalaris (Schinz, 1822) in *Fauna Ibérica* (ed. Salvador, A.) 390-407 (Madrid: Reptiles Museo Nacional de Ciencias Naturales, CSIC, 1998).
25. Pérez-Mellado, V. & Pérez-Cembranos, A. La fauna de la isla del Rey in *Medio Natural de la Isla del Rey* (ed. Fundació Olof Palme) 73-99 (Maó, Menorca, 2012).
26. Pérez-Mellado, V., Garrido, M., Ortega, Z., Pérez-Cembranos, A. & Mencía, A. The yellow-legged gull as a predator of lizards in Balearic Islands. *Amphibia-Reptilia* 35, 207-213 (2014).
27. Thompson, W.L., White, G.C. & Gowan, C. Monitoring Vertebrate Populations (ed. Academic Press, Inc.) (San Diego, 1998).
28. Fiske, I. & Chandler, R. unmarked: An R package for fitting hierarchical models of wildlife occurrence and abundance. *J. Stat. Softw.* 43(10), 1-23 (2011).
29. Borcard, D., Gillet, F. & Legendre, P. Numerical ecology with R (ed. Springer) (New York, 2011).
30. Legendre, P. & Fortín, M. Comparison of the Mantel test and alternative approaches for detecting complex multivariate relationships in the spatial analysis of genetic data. *Mol. Ecol. Resour*. 10, 831–844 (2010).
31. Oksanen, J. et al. Vegan: community ecology package. R package version 2.4-2. [https://CRAN.R-project.org/package=vegan](https://cran.r-project.org/package=vegan) (2017).

**Supplementary Figures and Appendices legends**

Supplementary Figure 1. Heatmaps based on genetic distances (*F*_ST_ values) among studied populations for first single SNPs (a) and for outlier SNPs (b). Heatmaps were obtained with ggplot2 package in R (<https://cran.r-project.org/web/packages/ggplot2/index.html>).

Supplementary Figure 2. DAPC analysis results with first single SNPs (a) and only outlier loci (b). Abbreviations: h, harbour; l, lighthouse.

Supplementary Figure 3. Plot of the number of new polymorphic loci added for each iteration of M=n for a subsample of our database using r80 method [7].

Appendix S1. The 72,846 first single SNPs in vcf format.

Appendix S2. The 1,355 outlier SNPs in vcf format.

**Supplementary Tables**

Supplementary Table 1. Values of genetic diversity parameters for 72,846 loci including nucleotide diversity (π), allelic richness (Ar), observed (H_o_) and expected (H_e_) heterozygosities and inbreeding coefficient (*F*_IS_), considering only variant positions and considering all positions in all *P. lilfordi* populations calculated using *populations* in *Stacks* and *hierfstat* in R.

|  | **Population** | **Private alleles** | **π** | | | **H_o_** | | | **H_e_** | | | ***F*_IS_** | | |
| --- | --- | --- | --- | --- | --- | --- | --- | --- | --- | --- | --- | --- | --- | --- |
|  |  |  | **Ar** | **Variant** | **All** | **Hierfstats** | **Variant** | **All** | **Hierfstat** | **Variant** | **All** | **Hierfstat** | **Variant** | **All** |
| Menorca | Aire | 52 | 1.1661 | 0.1661 | 0.0009 | 0.1534 | 0.1534 | 0.0009 | 0.1586 | 0.1586 | 0.0009 | 0.0328 | 0.0339 | 0.0002 |
|  | Colom | 6 | 1.1681 | 0.1681 | 0.0010 | 0.1523 | 0.1523 | 0.0009 | 0.1586 | 0.1590 | 0.0009 | 0.0395 | 0.0405 | 0.0002 |
|  | Porros | 945 | 1.1183 | 0.1200 | 0.0007 | 0.1282 | 0.1284 | 0.0007 | 0.1256 | 0.1141 | 0.0006 | -0.0203 | -0.0175 | -0.0001 |
|  | Rei | 20 | 1.1637 | 0.1638 | 0.0009 | 0.1575 | 0.1575 | 0.0009 | 0.1598 | 0.1548 | 0.0009 | 0.0147 | 0.0168 | 0.0001 |
| Cabrera | Cabrera( harbour) | 19 | 1.1818 | 0.1818 | 0.0010 | 0.1619 | 0.1619 | 0.0009 | 0.1717 | 0.1737 | 0.0010 | 0.0569 | 0.0573 | 0.0003 |
|  | Cabrera (lighthouse) | 3 | 1.1767 | 0.1767 | 0.0010 | 0.1603 | 0.1603 | 0.0009 | 0.1668 | 0.1646 | 0.0009 | 0.0390 | 0.0410 | 0.0002 |
|  | Esclatasang | 475 | 1.1655 | 0.1660 | 0.0009 | 0.1562 | 0.1563 | 0.0009 | 0.1596 | 0.1568 | 0.0009 | 0.0216 | 0.0233 | 0.0001 |
|  | Foradada | 746 | 1.1524 | 0.1533 | 0.0009 | 0.1421 | 0.1422 | 0.0008 | 0.1457 | 0.1444 | 0.0008 | 0.0247 | 0.0263 | 0.0001 |
| Mallorca | Dragonera | 4 | 1.1254 | 0.1269 | 0.0007 | 0.1064 | 0.1066 | 0.0006 | 0.1104 | 0.1092 | 0.0006 | 0.0365 | 0.0405 | 0.0002 |
|  | Colomer | 1 | 1.1219 | 0.1232 | 0.0007 | 0.1114 | 0.1115 | 0.0006 | 0.1133 | 0.1066 | 0.0006 | 0.0172 | 0.0229 | 0.0001 |
